# Imported Human West Nile Virus Lineage 2 Infection in Spain: Neurological and Gastrointestinal Complications

**DOI:** 10.3390/v12020156

**Published:** 2020-01-29

**Authors:** María Velasco, María Paz Sánchez-Seco, Carolina Campelo, Fernando de Ory, Oriol Martin, Laura Herrero, Octavio J. Salmerón Béliz, Teodora Minguito, Mª Carmen Campos, Francisca Molero, Alejandro Algora, Ana Vázquez

**Affiliations:** 1 Hospital Universitario Fundación Alcorcón, 28922 Madrid, Spain; mvarribas@gmail.com (M.V.); CCAMPELO@fhalcorcon.es (C.C.); omartin@fhalcorcon.es (O.M.); ojsalmeron@fhalcorcon.es (O.J.S.B.); mccamposm@fhalcorcon.es (M.C.C.); aalgora@fhalcorcon.es (A.A.); 2 Centro Nacional de Microbiología, Instituto de Salud Carlos III, 28220 Madrid, Spainfory@isciii.es (F.d.O.); lherrero@isciii.es (L.H.); teoml@isciii.es (T.M.); pmolero@isciii.es (F.M.); 3 Centro de Investigación Biomédica en Red en Epidemiología y Salud Pública (CIBERESP), 28029 Madrid, Spain

**Keywords:** West Nile virus lineage 2, human, Spain, neurological symptoms, digestive complications

## Abstract

We report the first human case of West Nile virus (WNV) lineage 2 infection imported to Spain by a traveler returning from Romania. Serum, cerebrospinal fluid and urine samples were analyzed and West Nile virus infection was identified by PCR and serological tests. The patient developed fever, diarrhea and neurological symptoms, accompanied by mild pancreatitis, described previously in very few cases as a complication of WNV infection and by alithiasic cholecystitis. Viral RNA was detected in urine until 30 days after the onset of symptoms and neutralizing antibodies were detected at very low titers. The phylogenetic analysis in a fragment of the NS5 gene of the virus showed a homology with sequences from WNV lineage 2 belonging to the monophyletic Central/Southern European group.

## 1. Introduction

West Nile virus (WNV) belongs to the *Flavivirus* genus in the *Flaviviridae* family. Humans, horses, and other vertebrate animals can acquire the infection through the bite of an infected mosquito, however, are considered as “dead-end hosts”. Although 80% of WNV infections are inapparent, about 20% of infected individuals develop fever with other symptoms such as chills, malaise, headache, backache, myalgias, arthralgias, gastrointestinal symptoms (nausea, vomiting, or diarrhea), and maculopapular rash. Around 1 in 150 infected people develop neuroinvasive disease, such as encephalitis, meningitis and acute flaccid paralysis [1]. Several atypical or rare presentations of WNV infection such as cerebellitis, myocarditis, hepatitis, pancreatitis, ocular manifestations, rhabdomyolysis and opsoclonus-myoclonus syndrome have been described in case reports or small case series [2,3,4,5,6]. 

Several WNV lineages have been described until now, but only two are pathogenic for humans (lineage 1 and 2). WNV lineage 1 (WNV-1) is widely distributed in America, Asia, Africa, Europe and Australia (Kunjin strains) and lineage 2 (WNV-2) is present in Africa and since 2004 in Europe [7]. The virus is considered emergent in Europe and neighboring countries in the last decade, with many cases in humans, birds and horses that have been reported [8,9]. In fact, a higher number of cases compared with transmission seasons in previous years were reported in the last epidemiological update in 2018 of WNV transmission season in Europe. In that year, the total number of reported autochthonous infections (*n* = 2083) far exceeded the total number of the previous seven years (*n* = 1832), showing an increase of 7.2% compared to 2017 [10].

In Romania the first outbreak of West Nile neuroinvasive disease (WNND) occurred in 1996, with 352 confirmed cases and 17 deaths due to WNV-1 [11]. After that, a national surveillance system was implemented, however only sporadic cases of WNND were diagnosed in humans. In 2010, a small outbreak of 49 cases occurred due to the introduction of WNV-2 similar to the strain causing an outbreak in 2007 in Volgograd, Russia. This strain belonged to the Eastern European clade, which had been detected in humans and mosquitoes in the following years [12]. Since then, the virus has not stopped spreading in Romania and in 2016, a third significant WNND outbreak in humans with 93 neurological cases was reported, due to a new WNV strain belonging to the Central/Southern European clade [13].

In Spain, WNV circulation in birds was confirmed for the first time in 2004 [14], and one human case was retrospectively diagnosed [15]. Similar sequences from WNV-1 were detected in birds (in 2007) and *Culex perexiguus* mosquitoes (in 2008) from Southern Spain [16]. Cases of WNV in horses and humans were reported in 2010 in the South of the country (Andalusia), which were caused by the WNV-1 strain [17]. Since then, WNV became endemic in Southern Spain, re-emerging every year and expanding northwards, causing outbreaks in horses, with further confirmed human cases in 2016 [18]. However, no data were available regarding the WNV lineage from the human cases. WNV-2 strain was detected for the first time in 2017 in Catalonia (Northern Spain) in goshawks by passive surveillance and no others reports have been done [19]. 

In this work we present and describe the first human infection of WNV lineage 2 imported from Romania to Spain showing important neurological and gastrointestinal complications. 

## 2. Materials and Methods

A Romanian 60-year-old male returned on September 7, 2018 from Adjud, Romania, a West Nile health alert zone. He was diagnosed as having a meningioma (treated with cranial radiotherapy years ago) and a thymoma that remained in clinical remission. Four days after his return, he began to have mild diarrhea and a fever of 38.5 °C He consulted at the Emergency Room on 13 September and a laboratory test showed a leukocyte count of 8610 cell/mL, hemoglobin level of 9.6 g/dL, platelet count of 131,000 cell/mL, mild coagulopathy and normal renal function. He was discharged with symptomatic treatment. Two days later, the patient consulted again because of the progression of intestinal symptoms and hypotension. He developed progressive neurological symptoms that evolved quickly: drowsiness, sleepiness, disorientation, aphasia, muscle weakness and a stiff neck; in addition, oral herpes lesions, progressive abdominal defense resembling an acute abdomen and hypotension developed in the following hours. At this time, laboratory results showed a leukocyte count of 12,030 cell/mL, hemoglobin level of 9.6 g/dL and a platelet count of 77,000 platelets/mL, 167 U/L of amylase and 1484 U/L of lipase, 13.6 mg/L of C-reactive protein, 0.33 ng/mL of procalcitonine and normal renal function. A chest X-ray showed an enlarged mediastinum (thymoma). Abdominal CT and cranial scans exhibited signs of alithiasic cholecystitis (thickening of the gallbladder wall), normal pancreas and uncomplicated (previously known) meningioma. A lumbar puncture was performed that yielded a transparent cerebrospinal fluid (CSF) sample with 404 cells/mL (77% neutrophils), 233 mg/mL of proteins, 57 mg/dL (96 in plasma) of glucose and 5.3 mmol/L of lactate. The patient was admitted to the critical care unit (CCU) on 15 September because of progressive neurological symptoms. Empiric treatment with an intravenous broad antibiotic spectrum (meropenem, ampicillin and vancomycin) and acyclovir was started. The patient quickly developed progressive respiratory insufficiency (respiratory distress and bilateral pleural effusion) requiring mechanical ventilation for up to 12 days. On the second day after admission the patient developed severe hypotension and oliguria with cardiac dysfunction consistent with Takotsubo Syndrome, requiring intensive fluid support and IV adrenaline, with progressive stabilization. After weaning mechanical ventilation and sedation withdrawal, lower limb paresis became prominent, consistent with moderate lower and upper sensory-motor polyneuropathy as by EMG at day 13 of admission. Structural CNS abnormalities were excluded by NMR imaging. The patient was discharged from the CCU to Infectious Diseases ward after 19 days of stay, with bradypsychia, lower limb paresia and a normal abdominal examination. At one month of hospital stay, the patient presented with paralytics ileus and after that abdominal pain due to mild pancreatitis (increased amylase levels and a lipase level of 3372 U/L), resolved in 10 days with conservative treatment. Patient was discharged to a rehabilitation facility on November 22, with hematological and biochemical alterations resolved, but the patient was still not able to walk by himself. One year later, the patient was fully recovered and the thymoma was surgically removed on 11 November 2019. The study was included in the diagnostic detection of WNV belonging to the Spanish National Program: Vector-borne diseases. The patient signed an informed consent for publication during their admission to the hospital.

The microbiological diagnosis was carried out in serum, CSF and urine samples taken at different days of disease progression using the BioFire^®^ FilmArray^®^ Meningitis/Encephalitis (ME) Panel (BioFire Diagnostic, BioMerieux, France). For the specific WNV diagnosis we used two different methods: a specific real-time PCR (qRT-PCR) able to detect all WNV lineages [20] and a generic RT-Nested-PCR for phylogenetic analysis [21]. To carry out the phylogenetic studies a fragment around 1000 nucleotides of the NS5 gene was sequenced. Comparisons with published sequences were performed in the NCBI BLAST database, and sequences were aligned by Clustal W in Mega 7 software [22]. The tree was created with the maximum likelihood method in PhyML [23], and the best fitting nucleotide substitution model was tested by Smart Model Selection using AIC (Akaike Information Criterion) [24]. Detection of IgM and IgG antibodies against WNV in serum and CSF samples was done by enzyme-linked immunosorbent assay (ELISA) based on capture and indirect methods, respectively (FOCUS, Cypress, California, CA, USA). To confirm the specificity of the antibody response, ELISA-positive samples were further tested by an in-house WNV-1, WNV-2 and Usutu virus (USUV) micro-neutralization test (VNT). Sera showing > 90% neutralization of the virus challenge were considered positive with a cutoff titer of 1:16. Vero cells and virus strains HU6365/08 (WNV-1), B956 (WNV-2) and SAAR1776 (USUV) were employed in the VNT. Additionally viral isolation in Vero E6 cell culture (ATCC) was set up with WNV RNA–positive urine samples at 10 and 20 days post onset of symptoms (dpo). Urine samples were diluted 1:10 in minimal essential medium with 2% fetal calf serum and 1 mL was inoculated onto a T25 flask of Vero E6 cell monolayers followed by incubation for 1 h. After incubation, 5 mL of minimal essential medium with 2% fetal calf serum was added to cells. Cell culture was incubated at 37 °C for 7 days and three blind passages were carried out. The presence of WNV in the culture fluids was checked for cytopathic effect (CPE) daily and by real-time RT-PCR. 

## 3. Results and Discussion

Results from molecular diagnosis in CSF for herpes virus 1, 2 and 6, cytomegalovirus, varicella zoster, enterovirus, paraechovirus, *Escherichia coli, Haemophilus influenzae*, *Neisseria meningitidis*, *Streptococcus pneumoniae*, *Listeria monoctogenes*, *Streptococcus agalactiae* y *Cryptococcus sp*. were negative. Blood culture, *Brucella* and HIV test were also negative. Extensive autoimmunity study (ANA by ELISA, ANCA by IFI and CCP, ACAG, ACAM by FIA) was negative and serum protein electrophoresis was normal. WNV genome was detected by qRT-PCR in serum at 7 dpo with a cycle threshold (Ct) value of 38 and in urine samples at 10, 20 and 30 dpo with Ct values of 30, 30 and 40, respectively. It should be noted that in urine the virus was detected with a higher viral load than in the serum sample and during more time, until 1 month after onset of the symptoms. These results reinforce the diagnosis algorithm described for WNV where the viremia of the WNV infection is relatively short (viral RNA can be detected in serum samples only around 7 dpo) but in urine samples viral RNA can be detected for a longer period and in higher copy numbers. This result has been observed before in several studies, in fact some of them have revealed the long persistence of WNV in urine particularly in patients with WN fever compared to patients with WN neuroinvasive disease, which had a higher viremia for a shorter period than those with WN fever [25]. Whether urine samples are a useful source for surveillance studies for WNV distribution and for analyzing the severity of the disease in patients with neuroinvasive and/or kidney manifestation needs to be investigated. Our findings reaffirm the utility of viral RNA detection in urine as a molecular diagnostic procedure for diagnosis of acute WNV infections, especially when acute samples are not available. WNV disease in this patient was confirmed by serology, either by the presence of WNV IgM antibodies in acute CSF and serum samples, IgG seroconversion at 20 dpo and neutralizing antibodies against WNV-2 were detected in sera samples since 45 dpo although at low titers (1/16 and 1/32). In a serum sample obtained 15 months after the onset of the symptoms, IgM antibodies were undetectable, but IgG antibodies and WNV-2 neutralizing antibodies were still detectable. Neutralizing antibodies against WNV-1 were detected below the cutoff titer of 1:16 in sera samples since 45 dpo. (Table 1). 

We attempted to isolate the WNV-2 strain detected in this patient from the two urine samples with a positive result in the qRT-PCR (Ct = 30) at 10 and 20 dpo. No cytotoxic effect was observed post-inoculation. We did not observe CPE in any of the three blind passages and we did not obtain viral genome amplification in the supernatant of the cell culture at 7 days post infection. In others studies WNV could be successfully isolated from urine samples and this finding indicates that infectious WNV may be excreted with urine but the relevance of WNV infectivity for virus transmission remains unknown [26]. In most cases, however, WNV isolation in cell culture has been unsuccessful and several causes have been proposed as the viral load of the sample, the characteristics of the urine samples and cell culture conditions.

**Figure 1:**
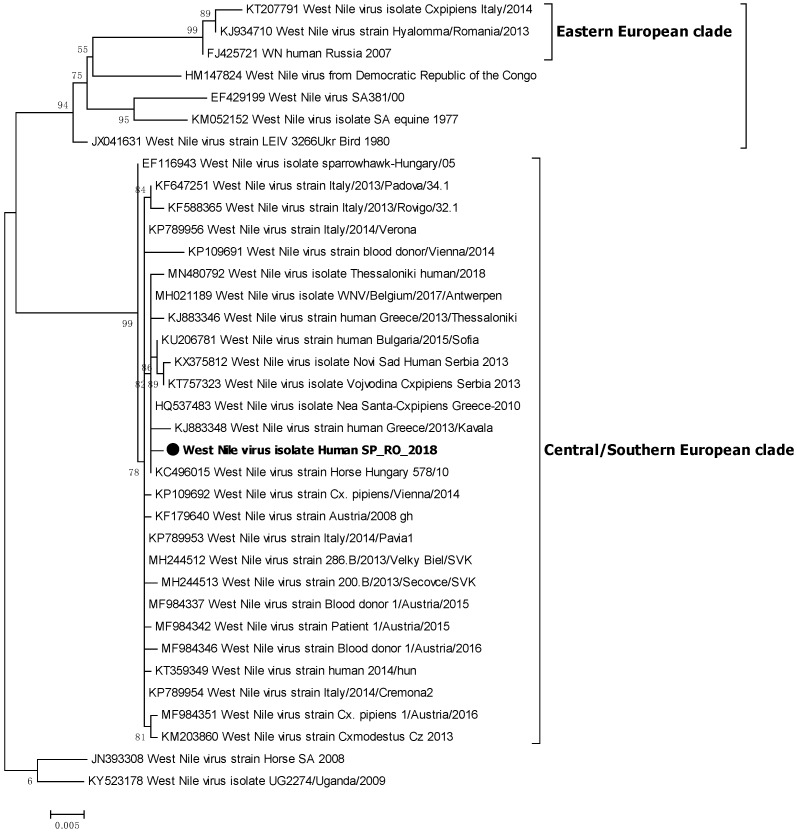
The tree was created with the maximum likelihood method in PhyML, based on 1032 nt of WNV-2 sequences available from NCBI GenBank (accession numbers provided). Black circle: sequence obtained in this study from human samples. GenBank accession number: MN966983. The TN93 + G model was used and the αLRT SH-like value was analyzed.

A maximum likelihood phylogenetic tree using the selected TN93 + G model was constructed and it showed that the WNV-2 strain amplified in this study clustered into the Central/Southern European WNV lineage 2 clade, which was detected in Romania for the first time in 2015 (Figure 1). In 2016, early amplification of the emerged WNV, and complete replacement in mosquito populations of the previously endemized virus in Southeastern Romania occurred and these events were associated with a human outbreak of severe WNV disease [13]. The result obtained in this work reflect that this strain is still circulating now in this country. The phylogenetic analysis shared the highest nucleotide similarity (over 99%) with several strains circulating in Greece, Belgium and Hungary in recent years. 

WNV-2 strains have been identified since 2004 in Europe being implicated in severe outbreaks. In 2018 human cases of WNV disease exceeded the total number from the previous seven years [8,9,10]. In this work, we described the first human case of WNV-2 reported in Spain. It was imported from Romania where 277 human cases and 43 deaths for WNV were reported during 2018. The patient had symptoms and signs of WNV meningoencephalitis, polyneuropathy and digestive complications such as alithiasic cholecystitis, mild pancreatitis and paralytic ileus. We cannot exclude critical ill neuropathy and sodium alteration as causes of polyneuropathy and paralytic ileus. Our case illustrates the importance of considering alithiasic cholecystitis and pancreatitis as manifestations of this infection and should be considered in patients with WNV infection who develop abdominal pain in the course of their disease. As far as we know, no cases of alithiasic cholecystitis in the course of WNV infection have been communicated previously. However, there are few reports with other flavivirus such as dengue, where it has been hypothesized that thickening of the gallbladder wall is produced by sudden plasma leakage rather than true inflammation [27]. Pancreatitis as a result of WNV infection has been described in the medical literature rarely. The first case was described in 1969 in Israel, in a 20-year-old woman [28]. In 1999 in New York, pancreatitis was an incidental finding discovered in the autopsy of an 80-year-old patient with meningoencephalitis and myocardial infarction [29]. In 2008 in Israel, the association between abdominal pain, high fever, neurological deterioration and CSF findings with elevated amylase and lipase levels and because no other causes of pancreatitis were found suggested an acute pancreatitis in an 88-year-old female induced by WNV [2]. Recently, in 2016 a 47-year-old Egyptian kidney transplant recipient developed a severe WNV infection with clinical neurologic involvement, thrombotic micro-angiopathy and pancreatitis, resulting in irreversible loss of kidney function [4]. All of these cases demonstrate pancreatitis as a rare complication of WNV infection, with the abdominal pain being very common so it should be considered in WNV suspected cases. Due to the few reports of patients with WNV-induced pancreatitis, it is difficult to establish the risk factors and further studies are needed to determine the frequency of this complication. 

This work reflects the sanitary importance that WNV represents in Europe nowadays, highlighting that infections caused by lineage 2 can also develop serious neurological clinical and also digestive complications such as pancreatitis and alithiasic cholecystitis. Awareness of this by health professionals is necessary for the detection of possible cases. 

## Figures and Tables

**Table 1:** Results obtained in the West Nile virus diagnosis. IND, indeterminate results; POS, positive; NEG, negative; IgM-Ab, IgM antibodies; IgG-Ab, IgG antibodies; NT, neutralization test; NT WNV-1, NT against WNV lineage 1; NT WNV-2, NT against WNV lineage 2; NT USUV, NT against Usutu virus; qRT-PCR, real time reverse transcription PCR.

Sample Type	Day after Onset of Symptoms (dpo)	qRT-PCR	IgM Ab	IgG Ab	NT WNV-1	NT WNV-2	NT USUV
CSF	7	NEG	POS (7.1)	NEG (0.1)			
Serum	7	POS (Ct = 38)	POS (8.7)	NEG (0.1)	NEG	NEG	NEG
Urine	10	POS (Ct = 30)					
Serum	10	NEG	POS (6.9)	NEG (0.5)	NEG	NEG	NEG
Urine	20	POS (Ct = 30)					
Serum	20	NEG	POS (6.4)	POS (2.4)	NEG	NEG	NEG
Urine	30	POS (Ct = 40)					
Serum	35		POS (6.1)	POS (3.1)	NEG	NEG	NEG
Serum	45		POS (7.7)	POS (3.9)	NEG	1/32	NEG
Urine	50	NEG					
Serum	99		POS (7.4)	POS (3.6)	NEG	1/32	
Serum	9 months		IND (1.3)	POS (3.3)	NEG	1/16	
Serum	15 months		NEG	POS (2.7)	NEG	1/32	
